# Virtual fracture clinic reduces patient X-ray volume for common wrist and ankle fractures

**DOI:** 10.1007/s11845-021-02812-y

**Published:** 2021-10-16

**Authors:** Conor S. O’Driscoll, Andrew J. Hughes, Fergus J. McCabe, Elaine Hughes, John F. Quinlan, Brendan J. O’Daly

**Affiliations:** Department of Orthopaedics, Tallaght University Hospital, Dublin 24, Ireland

**Keywords:** Ankle fracture, COVID-19, Trauma assessment clinic, TAC, Virtual fracture clinic, VFC, Wrist fracture, Trauma care, X-ray

## Abstract

**Background:**

Virtual fracture clinics (VFC) have been widely adopted worldwide as part of the changes in healthcare delivery during the COVID-19 pandemic. They have been shown to be a safe and effective method of delivering trauma care for injuries which do not require immediate intervention or specialist management, whilst maintaining high levels of patient satisfaction.

**Aims:**

Our aim was to evaluate whether VFCs reduce the volume of X-rays performed for common fractures of the wrist and ankle.

**Methods:**

A retrospective cohort review was performed. The pre-VFC group consisted of 168 wrist and 108 ankle referrals from March to September 2019. The VFC group included 75 wrist and 68 ankle referrals, during the period March to September 2020. The total number of X-ray images, carried out within a 3-month period for each fracture was summated, with statistical analysis performed following fracture pattern classification.

**Findings:**

A statistically significant decrease in mean X-rays was observed for isolated stable fracture patterns, such as non-displaced distal radius, − 0.976 (*p* = 0.00025), and Weber A ankle fractures, − 0.907 (*p* = 0.000013). A reduction was also observed for more complex fracture patterns such as dorsally displaced distal radius, − 0.701 (*p* = 0.129) and Weber B ankle fractures, − 0.786 (*p* = 0.235), though not achieving statistical significance.

**Conclusions:**

Virtual fracture clinics can reduce X-ray frequency for common stable wrist and ankle fractures, with resultant benefits for both patients and healthcare systems. These benefits may be sustained in patient care beyond the current COVID-19 pandemic.

## Introduction

Initially introduced in Scotland by the Glasgow Royal Infirmary Group [1], virtual fracture clinics (VFCs) have been widely adopted in orthopaedic departments both in Ireland and worldwide as part of the changes in healthcare delivery during the COVID-19 pandemic [2, 3]. They have been shown to be a safe and effective method of delivering trauma care for injuries which do not require immediate intervention or specialist management [4]. Furthermore, the majority of musculoskeletal injuries referred to orthopaedics are managed non-operatively [5], with a resultant large volume of fracture clinic attendances [6], making it challenging to accommodate patients in an expedited manner. VFCs have been shown to improve department’s compliance with British Orthopaedic Association Standards for Trauma and Orthopaedics (BOAST) guidelines for review of acute traumatic injury referrals within 72 h [7], while maintaining high levels of patient satisfaction, at over 95% reported across multiple studies [3, 8–10].

Our study’s aim was to evaluate the hypothesis that VFCs reduce the volume of X-rays carried out for common simple fractures of the wrist and ankle. This may prove beneficial, both to patients, through reduced radiation exposure, and to healthcare systems, by lowering financial cost and allowing alternate use of radiography services. We chose to use wrist and ankle fractures, as our study group. These are two of the most common orthopaedic injuries, with wrist fractures estimated to account for 17.5% and ankle fractures 9% of all fractures, [11] comprising a large clinical workload and are regularly managed via VFC [1, 12, 13].

## Methods

Our institution is a regional trauma centre with a catchment area of approximately 450,000 [14]. A consultant-led virtual fracture clinic was trialled in our institution prior to the COVID-19 pandemic and was quickly upscaled to become the primary interface between outpatient orthopaedics and the emergency department, from 24 March 2020. The VFC receives direct electronic referral from the emergency department. All cases are then discussed at the following scheduled VFC by a consultant orthopaedic surgeon and an extended spectrum physiotherapist (ESM), where the ED clinicians’ assessment and relevant images are reviewed. The patient is then contacted via a phone call by the ESM, advised regarding the outcome of their case discussion and given information regarding their injury. Patients may then be discharged, referred appropriately to a physiotherapist or occupational therapist led clinic, or requested to attend a physical fracture clinic. The VFC pathway is bypassed by patients whose case was discussed with on call orthopaedic services.

We carried out a retrospective cohort study, to investigate whether VFC reduced X-ray usage for common ankle and wrist fractures. We compared 276 patients referred from ED to the orthopaedic fracture clinic, during a period prior to the introduction of the VFC, to 143 patients who were referred via the VFC pathway. The pre-VFC group occurred from March to September 2019 and consisted of 168 wrist and 108 ankle referrals. The VFC group included 75 wrist and 68 ankle referrals, during the period March to September 2020.

Primary radiographs for each referral were reviewed independently by COD and FMcC to classify the injury based on predominant fracture pattern, to allow for comparison between the two groups, with the Danis Weber system used for ankle fractures involving the distal fibula. Cross-referencing with radiology reports was carried out where available. Instances of reviewer discordance were adjudicated by AH. The total number of X-ray images, included initial ED radiographs, carried out within a 3-month period for each injury was summated.

Statistical analysis was performed using Stata software version 16.1 (StataCorp, College Station, TX), to allow comparison of mean number of radiographs and estimate statistical significance. Following Shapiro–Wilk test of normality, *P* values were calculated using Welch *t*-test (2-tailed, unpaired assuming unequal variances). Following Bonferroni correction, a value of less than 0.00625 was used for statistical significance. Ethical approval for the project was granted by the hospital ethics department.

## Results

Patient demographics in terms of age and gender were similar between the two periods, with nil statistically significant difference between the populations (Table 1a, b).

The results for each of the most commonly observed fracture subtypes are shown in the tables below (Tables 2 and 3).

**Table.1:** Pre-VFC and VFC groups’ age (**a**) and gender (**b**)

**a **				
**Age**	**Pre-VFC mean X-rays (SD)**	**VFC mean X-rays (SD)**	**Welch t test**	**95% CI**
Wrist	53.52 (19.63)	50.67 (20.40)	*P* = 0.36	(− 8.97, 3.28)
Ankle	41.43 (16.61)	39.59 (15.27)	*P* = 0.50	(− 6.50, 3.21)
**b**				
**Gender**	**Pre-VFC (% female)**	**VFC (% female)**		
Wrist	72.28%	67.21%		
Ankle	53.19%	52.00%		

*SD* standard deviation

**Table.2:** The difference in wrist X-rays between the pre-VFC and VFC groups

**Wrist injury**	**Pre-VFC mean X-rays (95% CI) (Number)**	**VFC mean X-rays** **(95% CI) (Number)**	**Welch** ***t* test**	**Mean change** **(95% CI)**
No fracture	1.692 (1.238, 2.146) (n=13)	1.158 (0.916, 1.400) (n=19)	*P* = 0.037	− 0.534 (− 0.037, − 1.032)
Non-displaced	3.034 (2.798, 3.271) (n=58)	2.059 (1.637, 2.484) (n=17)	*P* = 0.00025*	− 0.976 (− 0.499, − 1.452)
Dorsal displacement	4.630 (4.069, 5.191) (n=27)	3.929 (3.162, 4.695) (n=14)	*P* = 0.129	− 0.701 (0.216, − 1.618)
Impacted	4.522 (3.955, 5.088) (n=23)	4.667 (4.125, 5.209) (n=6)	*P* = 0.679	0.145 (00.861, − 0.571)
Comminuted	6 (5.191, 6.809) (n=22)	4.5 (2.909, 6.091) (n=4)	*P* = 0.048	− 1.5 (-0.161, − 2.984)

*Statistically significant

**Table.3:** The difference in ankle X-rays between the pre-VFC and VFC groups

**Ankle injury**	**Pre-VFC mean X-rays (95% CI) (Number)**	**VFC mean X-rays** **(95% CI) (Number)**	**Welch *t* test**	**Mean change** **(95% CI)**
No fracture	1.692 (1.238, 2.146) (n=30)	1.281 (1.093, 1.470) (n=32)	*P* = 0.049	− 0.352 (− 0.0021, − 0.702)
Weber A Weber B	2.323 (2.005, 2.642) (n=34) 4.5 (4.012, 4.988) (n=30)	1.417 (1.197, 1.636) (n=36) 3.714 (2.331, 5.098) (n=7)	*P* = 0.000013* *P* = 0.235	− 0.907 (− 0.526, − 1.287) − 0.786 (0.621, − 2.192)

*Statistically significant

## Discussion

A statistically significant decrease in average X-rays was observed for isolated stable fracture patterns, such as non-displaced distal radius and Weber A ankle fractures. The natural history of these common fractures is well understood; they are highly unlikely to displace over time and have been shown to be safely managed via virtual fracture clinics [12, 13, 15]. Though suggestive, a statistically significant reduction was not demonstrated for more complex fracture patterns such as Weber B ankle and dorsally displaced distal radius fractures for which follow up imaging is often required.

Ankle sprains and Weber A ankle fractures have established evidence to be treated conservatively in a walking orthosis [16] and are suitable for virtual clinic management [15]. Bellringer et al.’s study of 309 radiologically stable isolated Weber B ankle fractures, demonstrated that they could be safely managed virtually, with 99.4% (307/309) achieving bony union using a standardised VFC management protocol that involves early full weight bearing in a supportive orthotic from first presentation in the ED, including 11 who were identified and proceeded to surgery. Consultant radiograph review ensured that 27 patients who were inappropriately referred with other injuries such as unstable Weber B on primary radiograph, Pilon fractures, and Bimalleolar fractures appropriately proceeded towards operative management [12].

White et al. successfully managed 1806 distal radius fractures using their virtual fracture clinic with no patient complaints reported. Early radiograph review allowed for stratification of patient care with un-displaced, minimally displaced or isolated extra-articular dorsally displaced (Colles) wrist fractures followed up in experienced nurse practitioner clinics, while other more complex wrist fractures would be seen in a physical fracture clinic between 5–8 days [13].

In our experience, virtual fracture clinic led to earlier primary radiograph review, case discussion, and formation of management plan by senior orthopaedic decision-makers, compared to traditional fracture clinics in which X-rays are regularly taken on arrival prior to surgeon review. This helped to reduce the number of unnecessary radiographs to which patients were exposed. Early virtual review also allows identification of cases which may require expedited review and possible operative intervention. In our study two injuries identified in VFC, proceeded towards operative management. This took place in a timely manner at an average of 11 days.

The average dose of an ankle or wrist X-ray is estimated to be 0.001–0.06 μSv [17]. Though low compared to other imaging modalities, this avoidable radiation dosage theoretically leads to an increased risk of cancer [18] and is often a source of concern for patients [19]. Manning et al. while surveying 946 consecutive patients attending a foot and ankle surgeon found regarding ankle X-rays, 55.9% of patients had thought about radiation exposure and 7.4% reported that they would potentially forgo an X-ray recommended by their doctor because they did not want the radiation exposure [19].

From a healthcare economics and resource allocation perspective, there is a significant cost of screening and time usage related to X-ray screening. The monetary cost of an ankle or wrist X-ray in our hospital is estimated to be 25 euro. The average time taken for a wrist X-ray is approximately 10 min and an ankle X-ray 15 min. A reduction in outpatient radiograph usage through the virtual fracture clinic pathway can facilitate a reallocation of manpower resources such as an additional radiographer available for theatre screening.

Furthermore, increased outpatient capacity created by virtual fracture clinics, which Kelly et al. in Connolly Hospital, Dublin, used to increase elective outpatient activity, reporting a 37% increase in elective clinic attendances and 25.7% increase in musculoskeletal injections performed during their study period [9].

From a patient perspective, reducing radiograph frequency is beneficial by reducing radiation exposure and inconvenient journeys to the hospital. This is particularly advantageous to patients with limb injuries, unable to drive who may have to rely on public transport, family, and friends to attend hospital VFC or radiology appointments [12].

Of utmost importance during the COVID-19 pandemic, VFC leads to a reduction in numbers attending physical fracture clinics and facilitates “social distancing,” limiting spread of disease [4, 20]. Pre-determined referral criteria ensure appropriate and consistent decisions in the ED, minimising the need for early senior opinion [21]. This can reduce the total time spent by a patient in the department [10], where they may also be vulnerable to COVID-19 transmission.

Future research may be directed towards the effects of virtual fracture clinics for other fracture types, and further studies with larger sample sizes which may demonstrate statistical significance for additional subtypes of wrist and ankle fractures.

## Conclusion

Virtual fracture clinics can reduce X-ray frequency for common stable wrist and ankle fractures, with resultant benefits for both patients and healthcare systems. These benefits may be sustained in patient care beyond the current COVID-19 pandemic.
